# Episodic memory improvement in illiterate adults attending late-life education irrespective of low socioeconomic status: insights from the PROAME study

**DOI:** 10.1590/1980-5764-DN-2023-0098

**Published:** 2024-06-28

**Authors:** Emma Patrice Ruppert, João Victor de Faria Rocha, Aída Lourandes da Silva, Kelle Luisa dos Santos Tomaz, Clarisse Vasconcelos Friedlaender, Joanna de Castro Magalhães Assenção, Luciana Paula Rincon, Norton Gray Ferreira Ribeiro, Dulce Constantina de Souza Santos, Ana Paula Zacarias Lima, Isabel Elaine Allen, Paulo Caramelli, Lea Tenenholz Grinberg, Francisca Izabel Pereira Maciel, Elisa de Paula França Resende

**Affiliations:** 1 Faculdade de Ciências Médicas de Minas Gerais, Belo Horizonte MG, Brazil.; 2 University of Pittsburgh, Pascoal Lab, Pittsburgh PA, USA.; 3 Universidade Federal de Minas Gerais, Faculdade de Medicina, Grupo de Pesquisa Neurologia Cognitiva e do Comportamento, Belo Horizonte MG, Brazil.; 4 Universidade Federal de Minas Gerais, Faculdade de Medicina, Programa de Pós-Graduação em Ciências Aplicadas à Saúde do Adulto, Belo Horizonte MG, Brazil.; 5 Escola Municipal Acadêmico Vivaldi, Belo Horizonte MG, Brazil.; 6 Escola Municipal Dr. Júlio Soares, Belo Horizonte MG, Brazil.; 7 University of California San Francisco, Global Brain Health Institute, San Francisco CA, USA.; 8 University of California, Memory and Aging Center, UCSF Weill Institute for Neurosciences, San Francisco CA, USA.

**Keywords:** Socioeconomic Factors, Cognitive Reserve, Memory, Episodic, Dementia, Literacy, Cognitive Aging, Fatores Socioeconômicos, Reserva Cognitiva, Memória Episódica, Demência, Alfabetização, Envelhecimento Cognitivo

## Abstract

The majority of people with dementia live in low or middle-income countries (LMICs) where resources that play a crucial role in brain health, such as quality education, are still not widely available. In Brazil, illiteracy remains a prevalent issue, especially in communities with lower socioeconomic status (SES). The PROAME study set out to explore basic education in illiterate adults as a means to improve cognitive reserve.

**Objective:**

This manuscript aims to explore the relationship between SES and learning, as well as cognitive outcomes, in an older illiterate population.

**Methods:**

This six-month clinical trial (NCT04473235) involved 108 participants, of which 77 concluded all assessments, enrolled in late-life basic education. SES assessments included Quality of Urban Living Index, Municipal Human Development Index and Household SES calculated for each participant. Cognitive assessments encompassed the Free and Cued Selective Reminding Test (FCSRT), a word list to assess reading, and the Beta III matrix.

**Results:**

The sample consisted primarily of women, with a mean age of 58.5. Participants improved their reading (p=0.01) and their FCSRT (p=0.003). Regarding episodic memory, women outperformed men (p=0.007) and younger participants improved more than their older counterparts (p=0.001). There was no association observed between SES and cognitive outcomes.

**Conclusion:**

Irrespective of SES, participants demonstrated positive outcomes after attending basic education. These findings highlight that late life education could be an important non-pharmacologic preventative measure, especially in LMICs.

## RESUMO

A maioria das pessoas com demência vive em países de baixa/média renda, onde recursos essenciais para a saúde cerebral, como educação de qualidade, ainda não são amplamente acessíveis. No Brasil, o analfabetismo ainda é frequente, especialmente em comunidades de baixo nível socioeconômico. O estudo PROAME teve como objetivo explorar a educação básica tardia em pessoas analfabetas como ferramenta para o aumento da reserva cognitiva.

**Objetivo:**

Investigar a relação entre nível socioeconômico com aprendizado e com desempenho em testes cognitivos, em adultos analfabetos.

**Métodos:**

Este estudo clínico de seis meses (NCT04473235) contou com 108 participantes inscritos no projeto Educação para Jovens e Adultos (EJA), dos quais 77 completaram os testes. O nível socioeconômico de cada participante foi medido usando-se: o Índice de Qualidade de Vida Urbana, o Índice de Desenvolvimento Humano Municipal e o nível socioeconômico doméstico. Avaliações cognitivas incluíram: o Teste de Recordação Seletiva Livre e Guiada (TRSLG), uma lista de palavras para avaliar leitura e a matriz Beta III.

**Resultados:**

A amostra era predominantemente feminina, com idade média de 58,5. Os participantes melhoraram a leitura (p=0,01) e o TRSLG (p=0,003). Com relação à memoria episódica, as mulheres tiveram resultados superiores aos dos homens (p=0,007) e participantes mais jovens melhoraram mais que seus colegas mais velhos (p=0,001). Não foi observada nenhuma relação entre o nível socioeconômico e o desempenho cognitivo.

**Conclusão:**

Independentemente do nível socioeconômico, participantes obtiveram resultados positivos após frequentar a educação básica. Isso sugere que a educação tardia pode ser uma medida preventiva não farmacológica importante, especialmente em países de baixa/média renda.

## INTRODUCTION

Approximately 57 million individuals are currently living with dementia, and projections indicate that this figure will triple by 2050^1-4^. Dementia is the primary cause of disability in high-income countries (HICs), but the majority of those affected reside in low or middle-income countries (LMICs)^4^. This discrepancy is primarily attributed to inequalities in access to resources that play a crucial role in brain health, such as quality education^1,2^. Notably, dementia can manifest up to a decade earlier in LMICs due to these inequalities^5^. Moreover, people with dementia who have low education manifest a more widespread cognitive impairment in comparison to those with higher schooling^6^. To mitigate the impact of these imbalances on brain health, it is imperative that they be addressed through targeted strategies, tailored to nations with lower economic means.

Approximately one third of Alzheimer’s disease cases can be attributed to underlying modifiable risk factors^4^. Control of the following factors can prevent as much as 40% of dementia cases: low education, hearing loss, hypertension, obesity, diabetes, alcohol abuse, traumatic brain injury (TBI), physical inactivity, depression, smoking, social isolation, and air pollution^2,3,6,7^. In Brazil, these factors account for up to 50.5% of dementia cases, especially low educational attainment and hearing loss^2,3^.

Due to the lifestyle-related nature of many preventable and protective factors, their impact is disproportionately felt in socioeconomically deprived regions^5^. Access to resources, including healthy diet, safety, education, healthcare services, physical activity, and even social factors like isolation and exclusion can pose significant challenges in communities with lower SES^2-4,8^. Paradoxically, much of the research into the role of these factors in dementia prevention has been conducted in HICs that do not face these same challenges^2,4,8,9^.

In 2022, 9.6 million Brazilians aged over 15 were illiterate, with 16% of the elderly population being illiterate^10^. Importantly, older adults with reduced literacy skills tend to have poorer health outcomes and an elevated risk of mortality^11^. Investigating prevention strategies tailored to individuals with low education levels is crucial for reducing the incidence and impact of dementia, especially in LMICs.

This work is part of a larger research project, the PROAME study, that aims to investigate the significance of acquiring basic education in later stages of life as a means to enhance cognitive reserve. This manuscript explores the relationship between socioeconomic status and learning in an older illiterate vulnerable population.

## METHODS

### Participants

This longitudinal clinical trial involved the screening of 130 adults who were actively participating in basic education classes offered by the Brazilian government under the program “Education for Teens and Adults” *(Educação de Jovens e Adultos)*. The inclusion criteria mandated participants to be illiterate (Test of Functional Health Literacy in Adults — TOFHLA score <53), and available to take part in the study for a minimum of 12 months^12-14^. Exclusion criteria included: significant cognitive complaints, unmanaged psychiatric conditions, ongoing substance abuse, evident cognitive impairment (defined as a score of ≤2 standard deviations for age and education on the Mini-Mental State Examination and/or a score of ≤6 on the delayed recall task from the Brief Cognitive Screening Battery), past medical history of dyslexia, contraindications to formal MRI procedures and MRI-detected structural brain lesions^15-19^.

**Figure 1:**
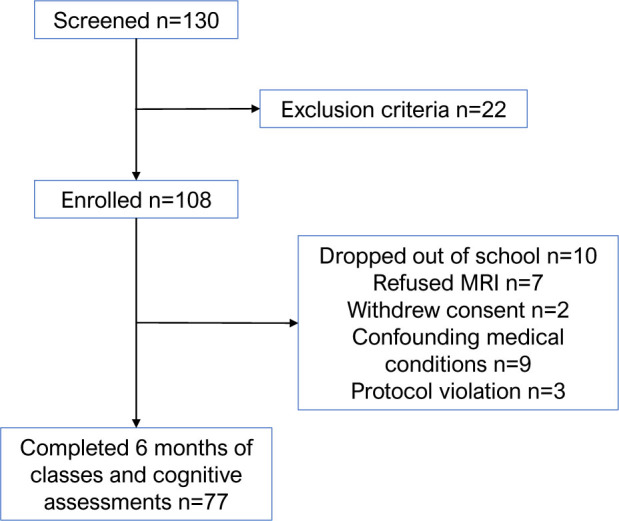
Flowchart depicting participants included in this study.

Following these criteria, a total of 108 participants were enrolled in the study. Data collection occurred at baseline and after six months of participation in basic education classes. At baseline, participants also answered the Cognitive Reserve Index questionnaire (CRIq)^20^. Figure 1 illustrates the flow of participants through the study, ultimately showing that 77 participants successfully completed all required clinical assessments and interviews both at baseline and after the educational intervention.

### Ethics

This study is part of a larger research program titled Better Memory with Literacy Acquisition Later in Life (NCT04473235) and was approved by the Ethics Committee of the Universidade Federal de Minas Gerais. Informed consent was obtained prior to data collection from all participants.

### Socioeconomic status assessment (SES)

#### Quality of Urban Living Index (QULI)

Created by the city of Belo Horizonte, this index serves as a comprehensive measure encompassing access to basic resources, cultural amenities, educational opportunities, sports facilities, housing conditions, urban infrastructure, environmental factors, healthcare services, public transportation and other urban services and security/safety within specific microregions of the city. These microregions make up 80 different areas. The index is available on the city hall’s official website and is graded on a scale from 0 (lowest) to 1 (highest). The most recent version is from 2016^21^. This assessment offers a valuable insight into the region. It is important to note that due to evolving social inequalities there is the possibility of misrepresentation, particularly for communities with lower SES situated within regions containing more affluent areas. This index was obtained from the 92 participants with verifiable addresses in Belo Horizonte. It revealed that these individuals were distributed across 20 different microregions within the city. This piece of data was missing for 16 participants.

The participants had an average score of 0.67, which is similar to the city-wide average of 0.63^21^. During the statistical analysis, the QULI score was treated as a continuous variable. However, for the purpose of discussion, we classified the measure using the median value of 0.67. This categorization resulted in a dichotomous distinction, for a more practical analysis — scores equal to or greater than the median were considered “high,” while scores lower than the median were considered “low”.

#### Municipal Human Development Index (MHDI)

A collaboration between the United Nations Development Program, the Brazilian Institute of Applied Economic Research, and the João Pinheiro Foundation, this index is made accessible through the interactive platform Atlas Brasil. It utilizes the Human Development Index (HDI) criteria: education, life expectancy, and gross national income per capita. This measure was collected for 92 participants. For statistical purposes this index was used as a continuous variable. During the discussion, we further divided the participants according to the HDI classification system as: very low (0–0.49), low (0.50–0.59), medium (0.60–0.69), high (0.70–0.79), to very high (0.80–1).^22^

A distinct advantage of this measure lies in its international recognition. However, the data used for its calculation is based on the last national census conducted in 2010, potentially rendering it less reflective of the current conditions.

#### Household Socioeconomic Status (SES)

The SES of each participant’s household was determined using Brazil’s Economic Classification Criteria. These criteria are nationally recognized and use a range of factors from a single household to estimate SES. These factors include quality of living conditions (such as number of bathrooms, electronic devices, automobiles, and household appliances), educational level of the primary provider, and availability of essential public services (access to treated water and paved roads)^23^. Each of these factors is assigned a point. These points are then tallied to yield a cumulative score that corresponds to a socioeconomic level — A, B1, B2, C1, C2, or D-E. Each level is associated with an approximate monthly income, in US dollars, of: $4,559 for level A, $2,092.54 for B1, $1,093.57 for B2, $610.53 for C1, $362.39 for C2, and $163.26 for D-E^23^. This data was missing for 19 participants, that did not complete the full assessment.

This tool offers the advantage of facilitating a tailored analysis of income and economic circumstances, providing a personalized insight into participants’ SES. However, this approach does not account for the way in which the area a person lives in can influence their access to essential services, safety, and overall socioeconomic standing.

### Cognitive assessments

Cognitive assessments were performed at baseline and after six months of education. The Free and Cued Selective Reminding Test (FCSRT), also referred to as delayed-recall episodic memory scores, was used for episodic memory^24,25^.

Reading skill evaluation was conducted by presenting the participants with a list of words devised by Rodrigues et al.^26^. This list consisted of 48 words to be read by the participants. Correctly pronouncing a word awarded a point, while an incorrect pronunciation resulted in zero points. Lastly, the Beta III matrix was employed to gauge non-verbal reasoning skills among the participants^27^.

### Intervention

The participants were categorized into two distinct groups based on their school enrollment. The control group (n=50) engaged in regular basic education classes, dedicating three hours each day for four days a week over a period of six months. The subjects covered in these classes included geography, mathematics, and history; however, these classes did not target literacy training. In contrast, the intervention group (n=58) attended, in addition to these basic education classes, intensive literacy instruction facilitated by a skilled educator, which was focused on how to read and write. Further characterization of these groups can be found in the Supplementary Material (https://www.demneuropsy.com.br/wp-content/uploads/2024/03/DN-2023.0098-Supplementary-Material.docx). This paper will not delve into the specifics of these subgroups, and this matter will be explored in another publication.

### Statistical analysis

The statistical analyses were carried out using the open software Jamovi Version 2.3.21.0. Descriptive and continuous variables were summarized using measures such as the mean, median, and interquartile ranges (IQR). Categorical variables were assessed in terms of frequency. During the analytical phase, QULI and MHDI were used as continuous variables. However, to provide further context, in the discussion, these SES measures were categorized into groups.

To examine the relationship between SES and TBI, simple linear regression analyses were conducted for QULI and MHDI, as well as Fisher’s exact test to determine if there was a significant association between Household SES and TBI. The exploration of potential associations between SES measures and improvements in episodic memory and reading skills was conducted using a pair of mixed linear models. The first model used the sum of attempts of FCSRT as the dependent variable. The model incorporated several covariates — QULI, MHDI, Household SES, Beta III, years of school attended as a child, age, sex, and timeline. The second model utilized the number of words read correctly and mirrored the covariates of the first model.

## RESULTS

The sample consisted of 108 participants, of which 77 successfully completed the cognitive assessments. The average age was 58.5 (standard deviation — SD=9.64). The majority were women (74.1%). In terms of race, most participants identified as black/brown, as shown in Table 1.

**Table 1.** Characteristics of the sample.

Characteristics	Frequency (%)
Sex	
Women	80 (74.1)
Men	28 (25.9)
Race*	
Black/brown	78 (88.6)
White	9 (10.2)
Indigenous	1 (1.1)
Mental health diagnosis	
Anxiety	33 (37.1)
Depression	18 (20.2)
Tobacco^†^	
Current use	6 (6.7)
Past use	40 (44.9)
Reported health conditions^†^	
Hypertension	46 (59.7)
Diabetes mellitus	22 (28.57)
Hearing difficulties	21 (27.27)
Attended school as a child^†^	
Yes	55 (61.8)
No	34 (38.2)
QULI^‡^	
Low	46 (50.0)
High	46 (50.0)
MHDI^‡^	
Medium	37 (40.2)
High	33 (35.9)
Very high	22 (23.9)
Personal SES^†^	
B	8 (9.00)
C1	14 (15.7)
C2	34 (38.2)
D-E	33 (37.1)

Abbreviations: QULI, Quality of Urban Living Index; MHDI, Municipal Human Development Index; SES, Social Economic Status.

Notes: *missing = 20; ^†^missing = 19; ^‡^missing = 16.

In terms of education, most participants (61.8%) reported attending school as a child and the average number of years of schooling, including during childhood and adulthood, was 1.9 years (SD=2.1). When questioned about their illiteracy, the primary explanations given by most participants were childhood labor (31.5%), followed by growing up in rural communities with limited school access (19.4%), learning difficulties (13.0%) and lack of parental encouragement (12.0%).

Regarding the CRIq scores in our sample, the mean was 72.5 (SD=6.8). When considering the different categories, the mean scores were as follows: education 76.6 (SD=10.6), work 87.1 (SD=6.4), and leisure 74.0 (SD=9.8). These scores are all in the medium-low category for cognitive reserve^20^.

Regarding SES, the participants’ QULI scores closely resembled the city-wide mean (M=0.67, SD=0.06, IQR=0.09), while the mean MHDI was categorized as high (M=0.73, SD=0.08, IQR=0.12), the Household SES revealed that most participants had a low income, as seen in Table 1.

Twenty-nine individuals reported having experienced a previous TBI, with an average of 27.9 years since the trauma occurred (SD=20.4, [0.16,60]). Information about previous TBI was missing for 19 participants. Approximately 41.4% of these individuals indicated seeking medical attention at a hospital following the injury, and twelve reported losing consciousness during the event. The results of linear regression analyses revealed that traumatic brain injury exhibited an association with lower QULI (ß=0.024, p=0.026, 95%CI 0.003–0.045) and a lower MHDI (ß=0.03, p=0.018, 95%CI 0.006–0.06). Fisher’s exact test showed an association between TBI and Household SES (p=0.046).

In respect to cognitive outcomes, after study completion participants exhibited a significant improvement in their episodic memory performance compared to baseline. However, this improvement was not different between control and intervention subgroups, which will be explored in another publication.

Notably, the factors of sex and age had significant effects on this improvement. Men demonstrated a lesser degree of improvement compared to women*.* Similarly, older participants displayed less improvement compared to their younger counterparts. In contrast, Beta III, years of school attended as a child, QULI, MHDI, Household SES did not have a significant impact on episodic memory improvement, as indicated in Table 2.

**Table 2.** Results of mixed linear regression on delayed-recall episodic memory scores.

	ß	p-value	95%CI
Timeline	3.122	0.003*	1.17; 5.08
Sex	-4.327	0.007*	-7.35; -1.30
Age	-0.178	<0.001*	-0.33; -0.03
Years of school attended as a child	0.096	0.740	-0.47; 0.66
Beta III	-0.154	0.645	-0.80; 0.49
QULI	7.673	0.463	-12.71; 28.05
MHDI	1.465	0.860	-14.80; 17.73
Household SES level			
B1–C2	-2.216	0.554	-9.52; 5.08
B2–C2	3.507	0.152	-1.26; 8.47
C1– C2	-0.126	0.947	-3.85; 3.59
D-E–C2	-0.402	0.786	-3.29; 2.48

Abbreviations: QULI, Quality of Urban Living Index; MHDI, Municipal Human Development Index; SES, Social Economic Status.

Note: *p<0.05.

Turning to participants’ reading abilities, an overall enhancement was observed after attending classes compared to initial testing. No other factors showed significant impact, as depicted in Table 3.

**Table 3.** Results of mixed linear regression on reading.

	ß	p-value	95%CI
Timeline	4.277	0.010*	1.14; 7.41
Sex	-7.45	0.184	-18.30; 3.40
Age	0.259	0.725	-0.27; 0.78
Beta III	1.621	0.179	-0.72; 3.96
QULI	-13.060	0.725	-85.51; 59.34
MHDI	51.120	0.088	-6.66; 108.90
Years of school attended as a child	-0.550	0.597	-2.58; 1.48
Household SES level			
B1–C2	8.686	0.164	-3.37; 20.75
B2–C2	6.686	0.110	-1.37; 14.74
C1–C2	2.69	0.415	-3.71; 9.09
D-E–C2	1.757	0.514	-3.71; 9.09

Abbreviations: QULI, Quality of Urban Living Index; MHDI, Municipal Human Development Index; SES, Social Economic Status.

Note: *p<0.05.

## DISCUSSION

**Figure 2:**
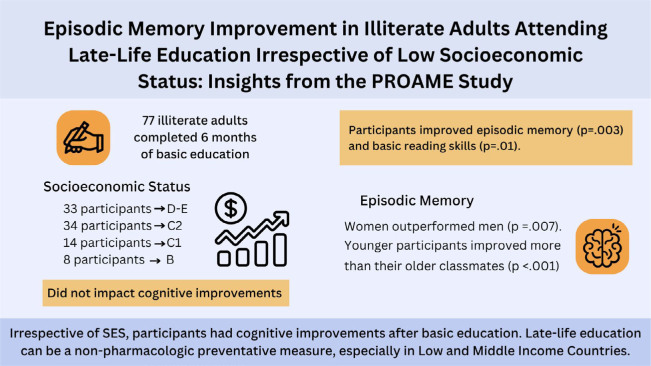
Visual abstract summarizing the study findings.

Disparities in the accessibility of preventive measures should contribute to the surge in dementia cases over the next three decades, particularly in LMICs^2,3,5^. While the protective influence of childhood education against dementia is well-documented, the potential impact of acquiring basic education later in life remains largely unexplored. This paper explored the role of SES in relation to learning in a sample of illiterate adults (Figure 2).

Our study findings showed significant improvement in both episodic memory and reading skills through late-life basic education. Secondly, age and sex were factors that affected episodic memory improvement. Lastly, our findings highlight that learning as an adult is an effective tool irrespective of low SES.

While our study primarily examined the impact of basic education on the brain health of illiterate adults, the findings are complemented by existing research on the role of continued education in late life. Notably, the Tasmanian Healthy Brain Project, a longitudinal study on cognitively unimpaired adults, demonstrated that individuals engaged in university-level schooling experienced greater improvements in verbal memory, verbal episodic memory, and episodic memory over a span of seven years compared to those who did not engage in schooling^28^. The novelty of our study lies in demonstrating similar benefits from basic education, suggesting that literacy programs tailored for illiterate adults can enhance brain health. This underscores the importance of offering public literacy programs for adults, especially in LMICs where illiteracy is still prevalent.

As expected, women had a greater improvement in episodic memory. Although the exact reason remains unclear, it is widely acknowledged that there exists a female advantage in verbal episodic memory^29,30^. The observed influence of age on episodic memory improvement also aligns with expectation, as age leads to a decline in fluid cognitive abilities. The decline typically commences around the age of 60 and accelerates in the subsequent decade^31^. Age might exert an even more pronounced impact than evident in our study, since the majority of participants were under the age of 70. This could indicate that public education programs for illiterate adults may be further optimized if started at an earlier age.

In regard to SES, our sample exhibited a seemingly high SES based on area of residency, such as QULI and MHDI, yet a low SES with regard to household income. Most participants fell within economic classes C1, C2 and D-E, corresponding to monthly incomes below the national income average of USD $632.21. This highlights that, despite residing in areas with available resources, participants had low personal SES, which could affect their utilization of these resources. Our findings indicate that education can benefit people from low and medium socioeconomic backgrounds alike. Additionally, our results showed an association between lower SES and previous TBI. And while the relationship between race and insurance status on TBI outcomes has been described, there is still little research exploring previous TBI and current SES, especially in Latin America^32,33^.

It is possible that the improvement seen in the participants was not solely due to education, but also due to the reduction in loneliness and in depression symptoms. While the role of loneliness and depression has been well documented as a risk factor for cognitive decline and dementia, there is still little research on how decreasing these factors could lead to better cognition^2-4,7^.

This study had a few limitations: a small sample size, potential for floor effects, as well as a relatively short time frame. Given that illiterate individuals often have lower overall SES, the sample may have been relatively homogeneous. This could limit the observed impact of SES on educational outcomes. Furthermore, due to the predominantly female composition of the sample, generalizing gender effects on a larger scale remains challenging. A notable challenge encountered was that some participants did not have a consistent attendance rate, which explains the difference in sample sizes depending on the day the data was collected.

This study offers valuable insights into the potential of late-life basic education as a public health strategy for addressing the global dementia burden, particularly in LMICs where illiteracy remains a significant concern. Importantly, it demonstrates that individuals from diverse socioeconomic backgrounds can benefit from learning interventions, especially if started at a younger age. These findings highlight an important non-pharmacologic preventative measure that could be very useful in LMICs. To strengthen these findings and deepen our understanding of the preventive role of late-life education in dementia, further investigations, specifically focusing on illiterate older adults, are warranted.
